# Comparison of Phenotypes of Headaches After COVID-19 Vaccinations Differentiated According to the Vaccine Used

**DOI:** 10.3390/vaccines13020113

**Published:** 2025-01-23

**Authors:** Carl Hartmut Göbel, Axel Heinze, Katja Heinze-Kuhn, Sarah Karstedt, Mascha Morscheck, Lilian Tashiro, Anna Cirkel, Qutyaba Hamid, Rabih Halwani, Mohamad-Hani Temsah, Malte Ziemann, Siegfried Görg, Thomas Münte, Hartmut Göbel

**Affiliations:** 1 Kiel Migraine and Headache Centre, 24149 Kiel, Germany; heinze@schmerzklinik.de (A.H.); khk@schmerzklinik.de (K.H.-K.); sarahkarstedt@gmail.com (S.K.); morscheck@schmerzklinik.de (M.M.); anna.cirkel@uni-luebeck.de (A.C.); hg@schmerzklinik.de (H.G.); 2 Department of Neurology, University Hospital Schleswig-Holstein, Campus Kiel, 24105 Kiel, Germany; 3 Department of Neurology, University Hospital Schleswig-Holstein, Campus Lübeck, 23562 Lübeck, Germany; thomas.muente@neuro.uni-luebeck.de; 4 Sharjah Institute of Medical Research, University of Sharjah, Sharjah 26666, United Arab Emirates; qalheialy@sharjah.ac.ae (Q.H.); rhalwani@sharjah.ac.ae (R.H.); 5 Department of Clinical Sciences, College of Medicine, University of Sharjah, Sharjah 26666, United Arab Emirates; 6 College of Medicine, King Saud University, Riyadh 12373, Saudi Arabia; mtemsah@ksu.edu.sa; 7 Institute of Transfusion Medicine, University Hospital Schleswig-Holstein, Campus Lübeck, 23562 Lübeck, Germany; malte.ziemann@uksh.de (M.Z.); siegfried.goerg@uksh.de (S.G.)

**Keywords:** coronavirus disease 2019, COVID-19, SARS-CoV-2 virus, vaccination, complications, headache, phenotype, postvaccinal headaches, head-to-head comparison, ICHD-3, Comirnaty (BioNTech), Jcovden (Johnson & Johnson), Sputnik V (Gamelaya), Covilo (Sinopharm), Spikevax (Moderna), Vaxzevria (AstraZeneca) and Convidecia (CanSino Biologics), spike protein, brain, mRNA vaccine

## Abstract

**Background/Objectives**: In this ongoing, multicenter, global cohort observational study, phenotypes of headaches after COVID-19 vaccination were directly compared between different vaccines. **Methods**: Phenotypes of postvaccinal headache were recorded in 18,544 participants. The study was launched immediately after the start of the global COVID-19 vaccination campaign on 12 January 2021 and continued until 1 August 2023. Specific aspects of headaches and related variables were collected via an online questionnaire. The clinical headache characteristics of patients vaccinated with the Comirnaty (BioNTech), Jcovden (Johnson & Johnson), Sputnik V (Gamelaya), Covilo (Sinopharm), Spikevax (Moderna), Vaxzevria (AstraZeneca), and Convidecia (CanSino Biologics) vaccines were investigated. **Results**: Across all vaccines, the median and mean latency of headache onset after vaccine administration were 12 h and 23.3 h, respectively. The median and mean headache duration were 12 h and 23.3 h, respectively. When the nonreplicating viral vector vaccine Sputnik V was used, headaches occurred the fastest, with a latency of 17 h. The latencies for the Vaxzevria and Convidecia nonreplicating viral vector vaccines were 14.9 h and 19.1 h, respectively. The Covilo inactivated whole-virus vaccine had a latency of 20.5 h. The latencies of the mRNA-based Comirnaty and Spikevax vaccines were 26.0 h and 22.02 h, respectively. Analysis of variance revealed no significant differences in the mean duration of postvaccinal headache for the vaccines tested. Compared with the Comirnaty, Covilo, and Vaxzevria vaccines, the Spikevax vaccine induced significantly greater headache intensities. Vaxzevria was associated with a significantly higher frequency of concomitant symptoms than the other vaccines. **Conclusions**: The phenotype of postvaccinal headache can vary significantly between vaccines. These results have clinical implications for differentiating between postvaccinal headache and other primary and secondary headaches. This knowledge is clinically relevant in differentiating life-threatening vaccination complications, such as thrombotic syndromes, which are also associated with headaches. Based on these results, new diagnostic criteria for postvaccinal headaches can be developed.

## 1. Introduction

Coronavirus disease 2019 (COVID-19) is an infectious disease caused by severe acute respiratory syndrome coronavirus 2 (SARS-CoV-2) [1]. COVID-19 was first detected in the Chinese metropolis of Wuhan [2]; it quickly spread and became an epidemic in the People’s Republic of China in January 2020 [3], eventually leading to a global pandemic [4,5]. No specific measures for active immunization were available at the beginning of the pandemic. During the second half of 2020, intensive work was being carried out worldwide to develop specific vaccines against COVID-19 [6,7,8,9]. Vaccination against COVID-19 has been proven to be the most important measure in preventing the spread of the disease [10,11,12,13,14,15,16,17,18,19].

The indication for COVID-19 vaccines is active immunization with the aim of preventing the disease [7,10,13,17,18,19,20]. The pathology of COVID-19 vaccines involves the immune system being exposed to one or more components of the SARS-CoV-2 coronavirus or being exposed to the virus as a whole [8], helping the immune system to subsequently build up protection against antigens [17]. COVID-19 vaccines differ in terms of their composition and the mechanisms by which the antigen or antigens come into contact with the immune system [21]. In 2022, worldwide, 66 vaccines were in the clinical development phase and 176 vaccines were in the preclinical development phase [22]. The modes of action, as well as the absolute and relative frequency distributions, of the different active substances are listed in Table S1 (Supplementary File S1) [23]. A summarized categorization of the different types of vaccines against COVID-19, their mechanisms of action, and their ingredients is given in Table S2 (Supplementary File S1) [8,22].

Temporary COVID-19 vaccine-induced reactions can occur during the development of the immune response [18,24]. Common vaccine-induced reactions include redness at the injection site, swelling, localized pain, headache, fever, fatigue, and flu-like symptoms. Vaccine-induced reactions are indications that the immune system is interacting with the vaccine, and these reactions are usually mild and temporary. However, reactions can also be very severe and persist for a long time or even permanently [25,26]. Vaccine-induced complications can have stressful and serious consequences for the people affected [27].

Acute or persistent vaccine-induced headache is the most common neurological complication associated with the COVID-19 vaccine [28,29,30,31,32,33,34,35,36]. This complication has not yet been classified by the International Classification of Headache Disorders (ICHD-3) [37], nor are there any internationally agreed-upon diagnostic criteria for this form of headache [8,16,23,32,33,34,36,38,39,40,41,42,43,44,45,46,47,48]. Additionally, COVID-19 vaccines are novel, previously unused substances, and the mRNA vaccines involve completely new modes of action for applied vaccinations [8,22,43]. In the authorization studies, only the number of headaches was recorded as a vaccine-induced reaction. However, detailed, clinical, qualitative phenotype reports of vaccine-induced headaches were not recorded. The characteristics of these headaches include the exact latency period between vaccine administration and the onset of headache, the duration, the character of the pain, the location of the pain, the accompanying symptoms, and possible pain-modulating factors.

The global vaccination campaign against COVID-19 enabled systematic investigations of these new forms of postvaccinal headache in very large national and international cohorts for the first time. Interim analyses of headache phenotypes after vaccination with the vector vaccine Vaxzevria^®^, formerly the COVID-19 vaccine AstraZeneca from AstraZeneca, and the mRNA vaccine Comirnaty^®^ from BioNTech/Pfizer have already been described [32,36].

In this study, the ongoing data analysis of postvaccinal headache phenotypes from 12 January 2021 until 1 August 2023 was extended. Additionally, the data from other vaccines covered were analyzed, and a head-to-head comparison of postvaccinal headache phenotypes was performed between the vaccines covered in this analysis: Comirnaty (BioNTech), Jcovden (Johnson & Johnson), Sputnik V (Gamelaya), Covilo (Sinopharm), Spikevax (Moderna), Vaxzevria (AstraZeneca), and Convidecia (CanSino Biologics). The aim of this study was to describe the detailed clinical characteristics of postvaccinal headaches among individuals who received the aforementioned vaccines in a large cohort. Accordingly, the quantitative and qualitative parameters of postvaccinal headache patients were examined via a head-to-head comparison.

## 2. Methodology

### 2.1. Study Design

Details of the headache phenotypes and related variables were collected via a publicly accessible online questionnaire. The questionnaire was available in several languages: English, German, Arabic, Japanese, Chinese, Hebrew, Lithuanian, French, and Russian. The questionnaire consists of 43 questions assessing the clinical characteristics of postvaccinal headaches. The questions are divided into the following groups:

- Type of vaccine received;
- Occurrence of postvaccinal headache;
- Possible headaches after previous vaccinations against other diseases;
- Temporal parameters of the headache;
- Headache localization;
- Headache characteristics;
- Headache intensity;
- Accompanying symptoms;
- History of headaches;
- Other comorbid diseases;
- Sociodemographic variables.

In accordance with a German ordinance on the right to receive the COVID-19 vaccination, people over 80 years old and residents and employees of care homes for elderly people were vaccinated first. Healthcare staff in hospitals, who were at very high risk of exposure to COVID-19, were also vaccinated as a top priority. As direct contact with vaccinated individuals was not possible due to anonymity, nursing home managers were contacted by email and asked to send information about the study to the residents and staff. A total of 12,000 nursing homes in Germany were contacted. Additionally, the departments responsible for organizing COVID-19 vaccination campaigns at university hospitals in Germany and the United Arab Emirates were contacted and asked to inform their staff about the study. The study was also publicized on the websites and social media platforms of the institutions. The present study analyzed the clinical characteristics of headaches among patients who were vaccinated with the Comirnaty (BioNTech), Jcovden (Johnson & Johnson), Sputnik V (Gamelaya), Covilo (Sinopharm), Spikevax (Moderna), Vaxzevria (AstraZeneca), or Convidecia (CanSino Biologics) vaccines and who completed the questionnaire from 12 January 2021 to 1 August 2023.

The Ethics Committee of the Faculty of Medicine at Kiel University approved this study (D403/21). The study was conducted in accordance with the 1964 Declaration of Helsinki and its subsequent revisions.

### 2.2. Data Collection

The clinical characteristics were recorded via an online database system. At the beginning of the questionnaire, the participants were informed that the data would be collected anonymously and that it would not be possible to withdraw from their participation in the study after submitting their answers. The participants could complete the questionnaire at any time after the onset of postvaccinal headache, and there was no time limit. As data collection began at the start of the vaccination campaign, the responses were generally related to the first vaccine dose. There was no differentiation in headache phenotypes between the first vaccination dose and subsequent boosters.

### 2.3. Bias and Missing Data

Compared with those in the general population, medical staff in tertiary university hospitals and people receiving care in nursing homes were over-represented in this study, as these individuals were in the highest-priority group for vaccines in Germany. The headache characteristics of people who did not voluntarily participate in this study could not be analyzed. Complete data were not available for all variables for all participants due to nonresponse; therefore, the number of responses may differ between individual analyses. Missing data were not included in this descriptive analysis.

### 2.4. Statistical Analysis

Continuous variables are expressed as the mean + standard deviation or as the median. Categorical variables are expressed as frequencies (%). Differences between two groups were examined via t tests. Differences between more than two groups were examined via analysis of variance (ANOVA) and the Scheffé post hoc test for pairwise comparisons. Differences between categorical variables were examined via the chi-square test. The statistical analyses were performed based on non-missing data. A significance level of 5% (alpha = 0.05) was considered statistically significant. The statistical analyses were carried out via SPSS 29.

## 3. Results

**Figure 1:**
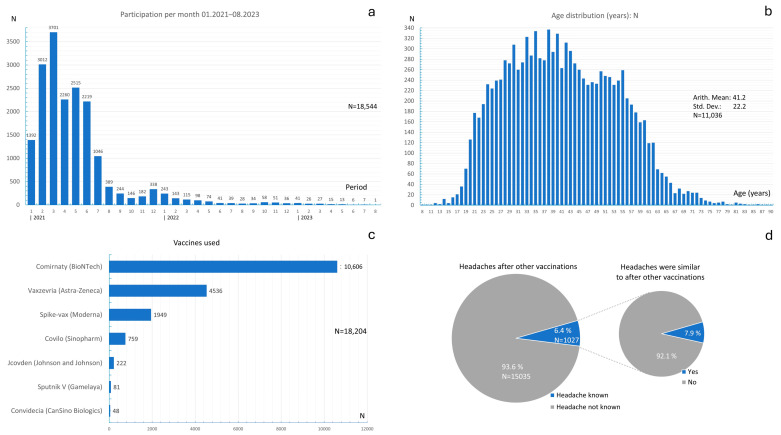
(**a**) Absolute frequency distribution of participation in the study depending on time; (**b**) absolute age distribution of participants across all vaccines; (**c**) absolute frequency distribution of the vaccines used; (**d**) frequency distribution of the occurrence of headaches with other vaccinations.

After the departments of university hospitals in Germany that were responsible for vaccination had been initially contacted and the management teams of nursing homes in Germany had been contacted, many vaccinated people completed the survey at the start of the vaccination campaign. Figure 1a shows the distribution of survey completion times. The majority of the responses were received in the first 7 months of the survey period. The month with the most responses was March 2021 (3701 respondents).

Figure 1b shows the age distribution of the participants across all vaccines. The mean age was 41.2 years ± 22.2 years, and ages ranged from 8 to 90 years.

Table S3 (Supplementary File S1) shows the means and standard deviations of age between the various vaccines. ANOVA revealed no significant difference in the age of the respondents between vaccines (ANOVA: *p* = 0.735).

A total of 19.8% of the sample were men, 80.1% were women, and 0.1% were nonbinary. The chi-square test revealed that there were sex differences between the vaccines (Table S4; chi-square test: *p* < 0.001 (Supplementary File S1)). Specifically, the results indicated that women were more likely to complete the survey when the Comirnaty, Spikevax, or Vaxzevria vaccines (approximately 80%) were used than when the Jcovden (68.5%) or Covilo (58.1%) vaccines were used. The high proportion of women can be explained by the high proportion of women in medical professions, especially in nursing professions.

The frequency distribution of the vaccines used is shown in Figure 1c. At the beginning of the vaccination campaign, the Comirnaty vaccine was predominantly used in Germany. Accordingly, this was the most frequently used vaccine across the entire study period (N = 10,606), followed by Vaxzevria (N = 4536) and Spikevax (N = 1949). Vaccines from China and Russia were used less frequently.

A total of 85.1% of the vaccinated individuals were vaccinated in the left upper arm, and 14.9% were vaccinated in the right upper arm. There was no significant difference between the vaccination sides in terms of the subsequent occurrence of postvaccinal headaches (chi-square *p* = 0.066).

A total of 16,788 people reported postvaccinal headaches. A total of 1696 people (9.2%) reported no postvaccinal headaches and were excluded from further analysis. This study was not designed to determine the prevalence of postvaccinal headaches associated with COVID-19 vaccination in relation to all vaccinations administered; rather, this study aimed to examine the headache phenotypes among individuals who reported postvaccinal headaches. However, as participation in the survey was open to all individuals, people who did not experience postvaccinal headaches were also able to complete the questionnaire; these individuals were excluded from further analyses.

Headaches after non-COVID-19 vaccinations were reported by 6.4% of the test subjects (Figure 1d), while 93.6% of the respondents reported that the COVID-19 vaccine was the first vaccine to induce a headache. Among the 6.4% of respondents who also experienced headaches after non-COVID-19 vaccinations, 92.1% stated that these headaches were not similar to those experienced after the COVID-19 vaccination, while 7.9% of these individuals reported that their previous headaches were similar to the headaches experienced after the COVID-19 vaccination.

**Figure 2:**
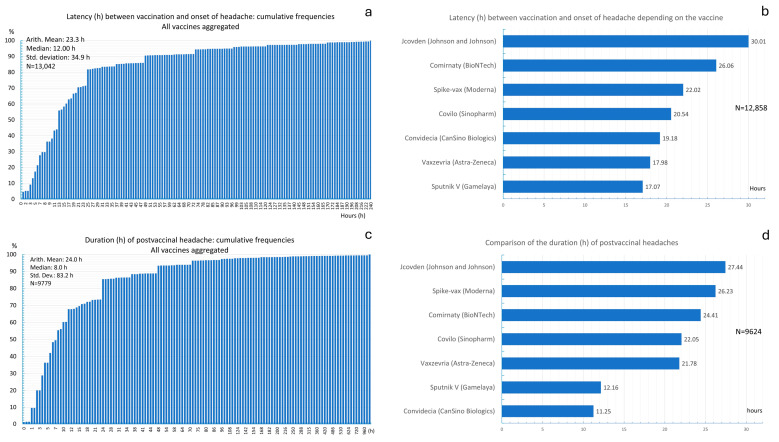
(**a**) Latency (in hours) between vaccine administration and the onset of postvaccinal headache across all vaccines; (**b**) comparison of latencies between vaccines; (**c**) cumulative frequencies of the duration of postvaccinal headache across all vaccines; (**d**) comparison of the mean duration of postvaccinal headache between vaccines.

Figure 2a shows the latency (in hours) between the administration of the vaccine and the onset of postvaccinal headache for all vaccines. The mean latency was 23.31 h, and the median latency was 12 h. This Figure shows that headaches could occur within the first hour after vaccination. In 50% of vaccinated individuals, headache occurred within 12 h of vaccination. In 90% of vaccinated individuals, headache occurred within 48 h of vaccination. Only a small proportion of respondents reported a longer latency between vaccine administration and the onset of headaches.

Figure 2b shows the head-to-head comparison of the latency between vaccines. When the Sputnik V nonreplicating viral vector vaccine was used, the latency was lowest (17 h). The longest latency between vaccination and headache onset was found for the Jcovden vaccine (30 h). Table S5 (Supplementary File S1) shows a detailed comparison of the latencies between the administration of the vaccine and the onset of headache between the vaccines. ANOVA revealed that the latencies were significantly different between vaccines (*p* < 0.001). The post hoc Scheffé test revealed that Vaxzevria had a significantly earlier onset of headache compared to Comirnaty, Jcovden, and Spikevax (Table S6 (Supplementary File S1)).

Figure 2c shows the cumulative frequencies of the duration of postvaccinal headaches. The average duration of postvaccinal headache among all vaccinated patients was 24.09 h, and the median duration was 8 h, indicating that 50% of the respondents had a headache duration of at least 8 h. In 90% of the participants, the postvaccinal headache subsided after 48 h, while 10% of respondents had longer headaches.

Figure 2d shows a comparison of the mean postvaccinal headache durations between vaccines. ANOVA revealed no significant differences in the mean duration of postvaccinal headache between vaccines (*p* = 0.657).

**Figure 3:**
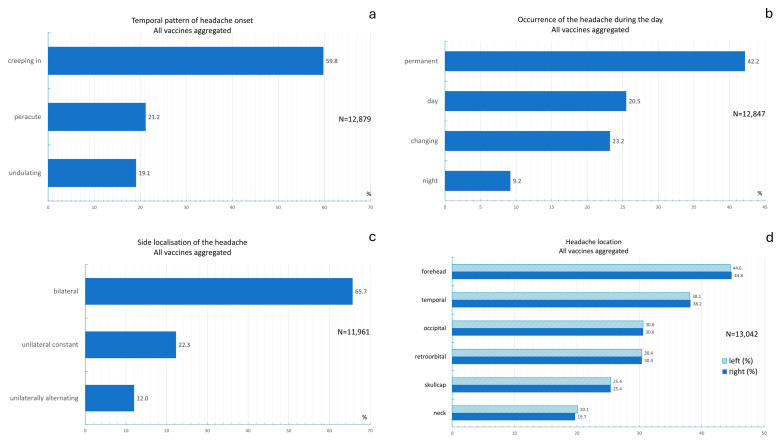
(**a**) Frequency distribution of the temporal pattern of headache onset; (**b**) frequency distribution of the preferred time of day of headache occurrence across all vaccines; (**c**) frequency distribution of side locations of headache occurrence across all vaccines; (**d**) frequency distribution of the lateralization of headaches across all vaccines.

Figure 3a shows the distribution of the temporal pattern of headache onset. Across all vaccines, 59.8% of the headaches had an insidious onset; 21.2% of the headache had a peracute onset; and 19.1% of the headaches developed gradually. The chi-square test revealed significant differences in the temporal pattern of headache onset between vaccines (chi-square test: *p* < 0.001). Table S7 (Supplementary File S1) shows the temporal patterns in headache onset between vaccines. The most common pattern for Vaxzevria was a gradual onset (62.3%). A peracute onset was most frequently reported for Convidecia (28.6%). An undulating onset of headache was most common for Covilo 35.9%.

The distribution of the most common time of day for the occurrence of headaches is shown in Figure 3b. The chi-square test revealed that the most common time of day for the occurrence of headaches differed significantly between vaccines (chi-square test: *p* < 0.001; Table S8 (Supplementary File S1)). Permanent headaches were most common with Spikevax vaccinations (46.8%). Daytime headaches were most common with Covilo (36.5%). Alternating headaches, regardless of the time of day, were most common with Jcovden (26.5%). Nocturnal headaches were most frequently reported for Sputnik V (25.6%).

The distribution of the lateral location of headache occurrence is shown in Figure 3c. The chi-square test revealed that the lateral location of headache occurrence differed significantly between vaccines (chi-square test: *p* < 0.001; Table S9 (Supplementary File S1)). Bilateral localization was the most common for all vaccines. Unilateral localization was the least common location, accounting for 17.3% of respondents who received Vaxzevria. Unilateral alternating headache localization was reported by only 2.8% of the participants who received the Sputnik V vaccine.

The distribution of the lateralization of headaches is shown in Figure 3d across all vaccines. The data revealed that postvaccinal headaches were most frequently felt in the forehead, temporal, occipital, and retro-orbital regions. Different lateral localizations were not evident when all the data were aggregated. The chi-square test revealed that the pain localization significantly differed between vaccines (chi-square tests: *p* < 0.001; Table S10 (Supplementary File S1)). Among individuals who received the Comirnaty, Jcovden, Spikevax, Vaxzevria, and Convidecia vaccines, headaches were most frequently located in the right or left forehead area. Among individuals who received the Sputnik V vaccine, headaches were most frequently reported in the retro-orbital region. Among individuals who received the Covilo vaccine, headaches were most frequently located in the cranial region.

**Figure 4:**
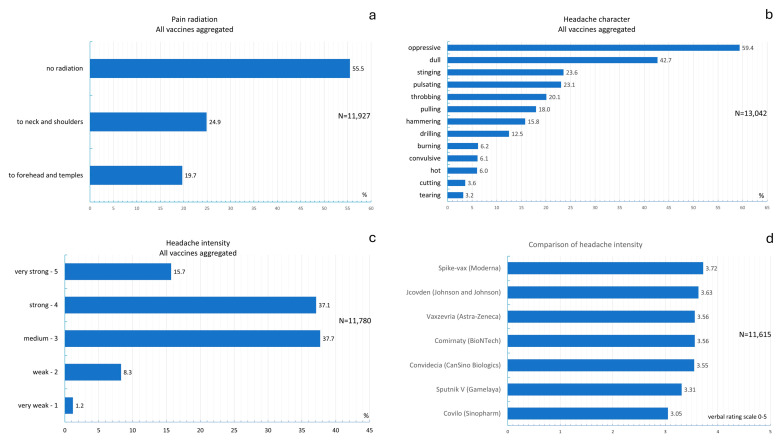
(**a**) Frequency distribution of pain radiation across all vaccines; (**b**) frequency distributions of headache characteristics across all vaccines; (**c**) frequency distribution of headache intensities aggregated across all vaccines; (**d**) analysis of headache intensity between vaccines.

The distribution of pain radiation is shown in Figure 4a. The chi-square test revealed that the radiation localization significantly differed between vaccines (chi-square test: *p* < 0.001; Table S11 (Supplementary File S1)). No radiation was the most frequent response among individuals who received the Comirnaty, Jcovden, Sputnik V, Covilo, Spikevax, or Vaxzevria vaccines. A total of 45.5% of the individuals who received Convidecia reported postvaccinal headaches that radiated to the neck and shoulders.

The distributions of headache characteristics for all vaccines are shown in Figure 4b. The most common headache characteristic was a pressing headache (59.4%), followed by a dull headache (41.7%). When headache characteristics were compared between vaccines, the chi-square test revealed a significant difference (chi-square test *p* < 0.001; Table S12). When the Convidecia vaccine was used, a stabbing headache was the most frequent characteristic; in contrast, a dull, pressing headache character was reported more frequently among individuals who had received other vaccines.

Figure 4c shows the distribution of headache intensities across all vaccines used. Headache intensity was assessed as follows: “no pain (0), very mild headache (1), mild headache (2), moderate headache (3), severe headache (4) and very severe headache (5)”. The most common headache intensity was “moderate” (37.7%), followed by “severe” (37.1%) and “very severe” (15.7%). A comparison of headache intensity between vaccines is shown in Figure 4d. The results revealed that the highest pain intensity was associated with the Spikevax vaccine (mean score of 3.72). The lowest pain intensities were associated with the Sputnik V and Covilo vaccines (means scores of 3.31 and 3.05, respectively). Analysis of variance revealed that there were significant differences in headache intensities across vaccines, as shown in Table S13 (ANOVA: *p* < 0.001 (Supplementary File S1)).

Table S14 (Supplementary File S1) shows the post hoc individual comparisons in the Scheffé test. Compared with the Comirnaty, Jcovden, Spikevax, and Vaxzevria vaccines, the Covilo vaccine induced significantly lower headache intensities. Compared with the Comirnaty, Covilo, and Vaxzevria vaccines, the Spikevax vaccine induced significantly greater headache intensities.

**Figure 5:**
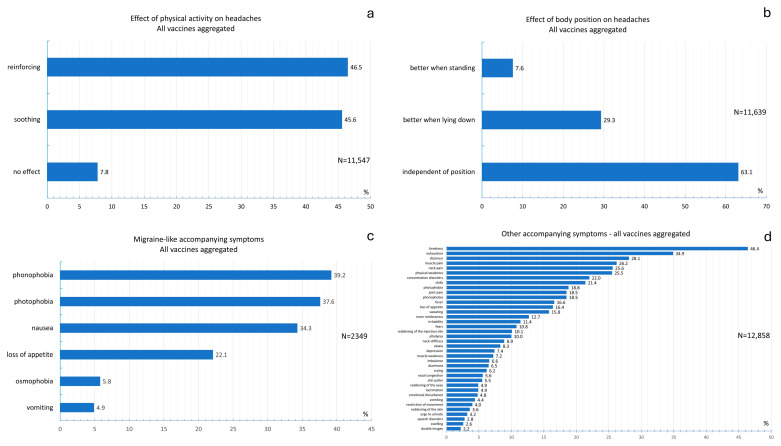
(**a**) Frequency distribution of the effects of routine physical activity on headaches across all vaccines; (**b**) frequency distribution of the effect of body position on headaches across all vaccines; (**c**) frequency distribution of migraine-like concomitant symptoms of headache across all vaccines; (**d**) frequency distribution of other concomitant symptoms, aggregated for all vaccines used.

The distribution of the effects of routine physical activity on headaches is shown in Figure 5a. The chi-square test revealed that the effects of routine physical activity significantly differed between vaccines, as shown in Table S15 (chi-square test; *p* < 0.001 (Supplementary File S1)). No effect of routine physical activity was the most frequent response among individuals who received the Comirnaty, Sputnik V, Covilo, or Convidecia vaccines. Headaches were more frequently aggravated by physical activity after vaccination with the Jcovden, Spikevax, and Vaxzevria vaccines.

Figure 5b illustrates the distribution of the effect of body position on headaches. Table S16 (Supplementary File S1) shows the distribution of the effect of body position between vaccines. The chi-square test revealed that there were significant differences in the effects of body position between vaccines (chi-square test: *p* < 0.001). Across all vaccines, participants most frequently stated that their headache was independent of their body position. When the Jcovden vaccine was used, 11.9% of the participants stated that the headache was better when they were standing, whereas when the Convidecia vaccine was used, 0% of the respondents stated that the headache was better when they were standing.

The distribution of migraine-like concomitant symptoms of headaches is shown in Figure 5c. Table S17 (Supplementary File S1) shows the distribution of migraine-like concomitant symptoms between vaccines. The chi-square test revealed significant differences in migraine-like concomitant symptoms between vaccines (chi-square test: *p* < 0.001). Phonophobia was reported most frequently among all vaccines. While phonophobia was reported at a frequency ranging from 35.7% to 40.6%, its frequency among individuals who received the Covilo vaccine was 16%.

The distribution of other concomitant symptoms across all vaccines is shown in Figure 5d. A total of 46.4% of respondents experienced fatigue, and 34.9% experienced exhaustion. More than 20% of the participants experienced chills, concentration problems, physical weakness, neck pain, muscle pain, and dizziness. More than 10% of the participants reported symptoms such as allodynia, redness at the vaccination site, anxiety, irritability, restlessness, sweating, loss of appetite, fever, phonophobia, joint pain, and photophobia. The frequencies of double vision, swelling, speech disorders, urinary urgency, reddening of the skin, restricted movement, vomiting, sensory disturbances, lacrimation, reddening of the eyes, pallor of the skin, nasal congestion, crying, diarrhea, balance disorders, muscle weakness, depression, ataxia, and neck stiffness ranged from 2% to 10%. The comparison of concomitant symptoms reported by more than 10% of respondents between vaccines is shown in Table S18 (Supplementary File S1). The chi-square test revealed that there was a significant difference in concomitant symptoms between vaccines (chi-square test: *p* < 0.001). For the Covilo and Convidecia vector vaccines, 18.5% and 21.4% of respondents reported fatigue after vaccination, respectively. In contrast, the frequency of fatigue was at least 45% among respondents who received the other vaccines. Chills were reported most frequently by 36.3% of individuals vaccinated with Vaxzevria. Fever was also most commonly reported among individuals who received Vaxzevria (30.7%). Compared with the other vaccines used, muscle pain, dizziness, and fatigue were most frequently reported among individuals who received Vaxzevria (35.3%, 29.3%, and 37.9%, respectively).

## 4. Discussion

### 4.1. Relevance

In this study, the phenotype of postvaccinal headache was recorded for all respondents across vaccines. Additionally, the phenotypes were analyzed and differentiated according to the vaccine used. This approach enabled a head-to-head comparison of vaccine-induced reactions in real-world situations. This type of comparison is important because completely new vaccines with completely new modes of action (e.g., mRNA vaccines) have been widely used internationally. Postvaccinal headaches are not included in the current edition of the international headache classification ICHD-3 [37]. However, the global COVID-19 vaccination campaign demonstrated that headache is the most common neurological complication of vaccination [28,29,30,31,32,33,34]. It also quickly became clear that life-threatening complications of vaccination, such as sinus vein thrombosis, stroke, or intracerebral hemorrhage, which can be caused by COVID-19 vaccines, can initially be heralded by headaches [49]. The headache phenotype has been identified as a key leading symptom in severe or even life-threatening complications of vaccination [28,29,30,31,32,33,34,49]. This finding made the study data particularly clinically relevant, as it was unclear how these headaches could be differentiated from other common headaches as a vaccine-induced reaction—the phenotype and the most important characteristics of these headaches were not known. The temporal course of headaches after COVID-19 vaccination and of headaches with other neurological complications had become an important diagnostic marker. However, it remained unclear how the very common postvaccinal headache can be differentiated from headaches as a symptom of rare severe vaccination complications.

Due to the lack of detailed knowledge about the phenotypes of postvaccinal headaches, it was not possible to differentiate postvaccinal headaches from headaches as a symptom of rare severe vaccination complications during the first few months of the vaccination campaign. This phenomenon led to uncertainty, anxiety, and many presentations in emergency outpatient clinics, inducing financial burdens. The phenotype of postvaccinal headaches has not been previously elucidated from a scientific point of view.

The majority of the data were collected in the first 8 months of the COVID-19 vaccination campaign. The differences between initial vaccinations and subsequent boosters were not examined. In this study, only data on the vaccines that were used by the participants were analyzed.

This study was not designed to determine the prevalence of postvaccinal headaches after COVID-19 vaccination in relation to all vaccinations administered. To do this, a population of test subjects would have had to have been asked whether headaches had occurred after vaccination. However, the frequency of postvaccinal headaches after vaccination against COVID-19 was known from authorization studies [50,51,52,53,54,55,56,57,58,59,60,61,62,63,64,65,66,67,68,69,70,71,72,73,74,75,76]. The central aim of this study was to perform a detailed examination of the previously unknown phenotypes of postvaccinal headaches.

### 4.2. Headaches with Other Vaccinations

The analysis revealed that 93.6% of the participants reported that they had not experienced headaches after non-COVID-19 vaccinations; only 6.4% stated that they had also experienced headaches after other vaccinations. Among the latter group of patients, 92.1% reported that the headache experienced after vaccination against COVID-19 was not similar to the headache experienced with non-COVID-19 vaccinations. These findings indicate that, compared with headaches after other vaccinations, headaches after COVID-19 vaccination have a differentiable phenotype.

### 4.3. Time Parameters

Across all vaccines analyzed in this study, postvaccinal headaches began at a mean interval of 23.3 h after the vaccine was administered. The median duration of postvaccinal headaches was 12 h. This finding indicates that 50% of respondents experienced postvaccinal headaches within 12 h. Given that participants still reported the onset of headaches several days after vaccination, the analysis of the cumulative frequency provides a detailed understanding of the distribution of the latency between vaccine administration and the onset of headaches. After approximately 20 h, postvaccinal headaches occurred in approximately two-thirds of respondents. Approximately 90% of the participants experienced postvaccinal headaches within 48 h of vaccine administration. The close temporal relationship between vaccination and the occurrence of postvaccinal headaches is therefore an important differential diagnostic feature in determining whether a headache was induced by vaccination.

The time to the onset of postvaccinal headache differed significantly between vaccines. Headaches occurred most rapidly after vaccination with the Sputnik V and Vaxzevria vaccines (means of 17.0 and 17.9 h, respectively). Both the Sputnik V [51,61,63] and Vaxzevria [56] vaccines are vector vaccines that carry the gene encoding the spike protein of SARS-CoV-2. These vaccines are technologically similar. By making the entire finished gene for the SARS-CoV-2 spike protein available, it is hypothetically conceivable that the immune reaction will start more quickly; thus, postvaccinal headaches will occur earlier as a vaccine-induced reaction than with component vaccines. Hypothetically, an earlier onset can also be explained by the initial strength of the immune response, which can occur with different vaccines.

The mean duration of postvaccinal headaches across all vaccines was 24 h. The median duration was 8 h, meaning that 50% of respondents had a headache duration of 8 h. Only 10% of respondents had postvaccinal headaches lasting longer than 48 h.

An important temporal characteristic of postvaccinal headaches is the mean interval of 23 h between vaccine administration and the onset of headaches. The time between vaccine administration and the onset of headache can vary from approximately 17 h (for Sputnik V) to approximately 30 h (for Jcovden). The duration of postvaccinal headache can also vary from approximately 11 h (for Convidecia) to 27 h (for Jcovden).

Approximately 60% of the respondents reported that their postvaccinal headaches began slowly and gradually. The headaches occurred peracutely in approximately 21% of cases. Gradual onset was most common among individuals who received Vaxzevria (62%). Sudden onset was most common among individuals who received Convidecia (28.6%).

Across all vaccines, 42.2% of the participants reported a permanent headache phenotype throughout the day. Headaches that occurred mainly during the day were reported by 25.5%, and headaches that occurred mainly at night were reported by 9.2%. Permanent headaches were reported most frequently among individuals who received the Spikevax vaccine (46.8%). A precise pattern regarding the preferred time of day of the occurrence of headaches could not be identified.

### 4.4. Localization

A total of 65.7% of respondents reported bilateral postvaccinal headaches across all vaccines. This effect was most pronounced for the Vaxzevria vaccine (73.2%). However, unilateral alternating or unilateral constant headaches were also reported across all vaccines.

Across all vaccines, headaches were most frequently located in the forehead, temporal, occipital, and retro-orbital regions. There was no difference in lateral localization. For Sputnik V, retroorbital localization was reported most frequently at 37.2%. When the Covilo vaccine was used, headaches were most frequently localized in the cranial region (29.4%). For the other vaccines, the headache was most frequently located in the right or left forehead area.

In terms of the aggregated data for all vaccines, 55.5% of the respondents stated that the pain did not radiate. In 24.9% of the patients, the headaches radiated to the neck and shoulders, and in 19.7% of the patients, they radiated to the forehead and temples. Radiation to the neck and shoulders was most common in 45.5% of those vaccinated with Convidecia. In comparison, the other vaccines most frequently did not cause any radiation-related pain.

### 4.5. Pain Characteristics

The aggregated data for all vaccines revealed that the most common type of pain was pressing (59.4%) or dull (42.7%). When vaccinated with the Convidecia vaccine, 28.6% of those vaccinated most frequently reported pulling pain. With the other vaccines, the most common pain characteristic described was dull.

### 4.6. Pain Intensity

When the data were aggregated across all vaccines, the intensity of pain was moderate (37.7%) or severe (37.1%). A total of 15.7% reported very severe pain. The lowest headache intensities were found when Covilo was used for vaccination (3.05 scale divisions), and the highest headache intensity was found when Spikevax was administered (3.72 scale divisions).

An analysis of the aggregated data across all vaccines revealed that physical activity could increase headaches in 46.5% of patients and alleviate headaches in 45.6% of patients. The influence of physical activity on headache intensity is therefore not clearly distinguishable as a diagnostic feature.

Headache intensity was not influenced by body position in 63.1% of the patients. A total of 29.3% reported an improvement in pain when lying down. A comparison of active substances showed that headaches most frequently occurred independently of body posture with all vaccines.

### 4.7. Accompanying Symptoms

An analysis of the individual active vaccines revealed that dizziness was the most frequently reported concomitant symptom when Convidicea were used (28.6%). For other vaccines, however, tiredness and fatigue are documented as the most frequent accompanying symptoms. When Vaxzevria was administered, numerous concomitant symptoms occurred with a significantly greater frequency than with the other vaccines. This applies to fever (30.7%), joint pain (27.0%), chills (36.3%), concentration problems (21.4%), muscle pain (35.3%), dizziness (29.3%), and fatigue (37.9%). Compared with other vaccines, Vaxzevria had the most documented side effects in terms of quality and quantity.

There are clear differences in relation to the Vaxzevria vaccine with respect to concomitant symptoms. Compared with other vaccines, the most extensive side effects were documented for this vaccine, both qualitatively and quantitatively. For the Covilo and Convidecia vector vaccines, 18.5% and 21.4% of participants reported fatigue after vaccination, respectively. In contrast, fatigue was found in more than 45% of patients receiving other vaccines. Chills were reported most frequently by 36.3% of those vaccinated with Vaxzevria. Fever was also the most common symptom of vaccination with Vaxzevria (30.7%). Muscle pain, dizziness, and fatigue symptoms were also more common after vaccination with Vaxzevria than with the other vaccines used (35.3%, 29.3%, and 37.9%, respectively).

In pivotal clinical trials with Vaxzevria, the use of prophylactic paracetamol prior to vaccination was recommended in all trials (except in the COV005 trial; it was introduced as an amendment during the COV001 trial) [57]. Vaccinees were advised to continue taking 1000 mg of paracetamol at 6 h intervals for 24 h to reduce the side effects caused by the vaccine. In the COV001 study, the effect of paracetamol on immunogenicity was investigated in participants via a standardized ELISA [70]. When the study participants who received paracetamol were compared with those who did not, no differences were found in the generation of antispike responses. However, it is possible that the headache phenotype and incidence of postvaccinal headache in these studies were attenuated by the prophylactic administration of paracetamol. No prophylactic administration of paracetamol took place in the open application as part of the vaccination campaign. In Germany, the prophylactic use of paracetamol was not recommended and was not included in the Robert Koch Institute (RKI) information sheets. It is also not included in the package leaflet. The headache phenotype recorded here can therefore provide a more realistic, unobscured picture for the first time.

Cases of cerebral venous thrombosis and other unusual thrombotic events have occurred following vaccination with the Vaxzevria vaccine [31,32,54,77,78,79,80,81]. This has led to widespread age restrictions in many countries worldwide, including the complete cessation of the use of the Vaxzevria vaccine. Headache is the leading symptom of cerebral venous thrombosis. For the differential diagnosis of headache due to this vaccine and headache due to cerebral venous thrombosis, it is of central clinical importance, as explained, to investigate whether and how the phenotypes and courses of these headaches can be differentiated. AstraZeneca’s COVID-19 vaccine Vaxzevria was withdrawn from the market due to a significant drop in demand. The decision to withdraw the marketing authorization was requested by AstraZeneca itself for commercial reasons and came into effect on 8 May 2024.

### 4.8. Classification and Differential Diagnosis

**Figure 6:**
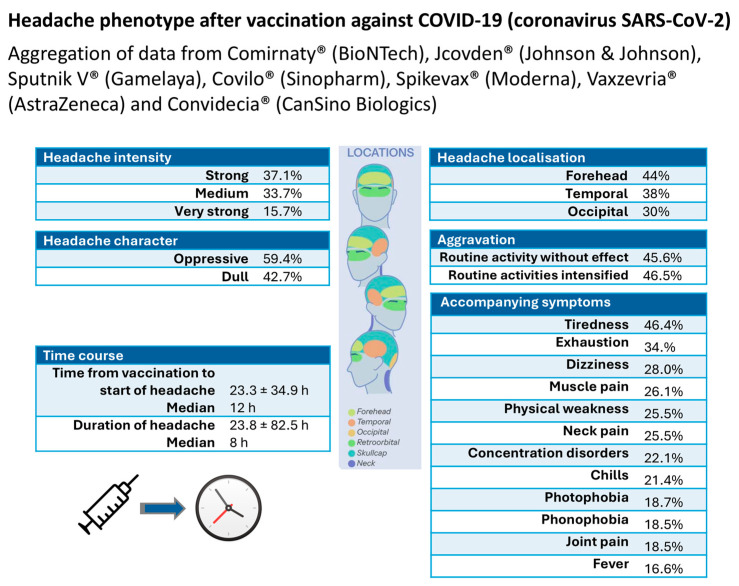
Headache phenotypes associated with vaccination against COVID-19 when the data of all the vaccines analyzed were aggregated.

The International Headache Classification does not yet provide operationalized criteria for the classification and diagnosis of postvaccinal headaches, whether in general or specifically for postvaccinal headaches after vaccination against COVID-19. On the basis of the data collected, the diagnostic criteria of the ICHD-3 [37] were proposed for headaches attributable to vaccination against COVID-19 [32]. Figure 6 summarizes the headache phenotype associated with vaccination against COVID-19 when the data for all the vaccines analyzed were aggregated.

Knowledge of these diagnostic criteria can be crucial, as postvaccinal headaches can also occur as secondary headaches in patients who have no other headache disorder. While headaches associated with a systemic viral infection typically have no specific headache characteristics in terms of temporal aspects, pain characteristics, localization, or accompanying symptoms [37], the results of this study reveal a specific headache phenotype with characteristic accompanying symptoms. It is important to differentiate postvaccinal headaches from life-threatening complications of vaccination, such as sinus vein thrombosis, stroke, or intracerebral hemorrhage, which may initially be heralded by headaches [49]. The phenotype of the headache serves as a central guiding symptom to detect or rule out a serious or even life-threatening complication of the vaccination [28,29,30,31,32,33,34,49].

### 4.9. Headaches with Other Vaccination Complications

Garcia-Azorin et al. [31] compared the temporal occurrence of headaches after COVID-19 vaccination in relation to cerebrovascular events. They analyzed all consecutive events recorded in the United States Vaccine Adverse Event Reporting System for events following COVID-19 vaccination in the period from 1 January to 24 June 2021. They analyzed the timing of the occurrence of postvaccinal headache in patients who experienced no adverse events or cerebrovascular events, including cerebral venous thrombosis, ischemic stroke, or intracranial hemorrhage. A total of 314,610 events occurred during 306,907,697 COVID-19 vaccinations. A total of 41,700 headache events and 178 cerebrovascular events were recorded. If only isolated headaches occurred, then the median time between the administration of the vaccine and the onset of the headache (1 day) was shorter than that for headaches associated with cerebral sinus vein thrombosis (4 days). In the case of ischemic stroke, the latency was 3 days; in the case of intracranial hemorrhage, it was 10 days. If headaches occurred within 1 day after COVID-19 vaccination, they were very rarely associated with cerebrovascular events (0.4%). The delayed onset of headache 3 days after the administration of the vaccine was a precise diagnostic biomarker for the occurrence of concomitant cerebrovascular complications. A latency of more than 3 days between the administration of the vaccine and the onset of headache was observed in 87.1% of respondents, which is specific to vaccine-associated cerebrovascular events. If headache occurred within 3 days after vaccination, then the occurrence of cerebral venous thrombosis, ischemic stroke, or intracranial hemorrhage could be excluded, with probabilities of 99.98%, 99.86%, and 99.95%, respectively (negative predictive value). Headaches occurred within one day in respondents who had no subsequent vascular complications (range 0–1 day). These data clearly support the findings of our study. Patients who experienced cerebral venous thrombosis experienced headaches an average of 3 days after receiving the Comirnaty vaccine, 5 days after receiving the Moderna vaccine, and 10 days after receiving the with Jcovden vaccine (*p* < 0.001). Interestingly, as in our study, vaccination with Jcovden resulted in a longer latency between vaccine administration and the onset of headache, as well as a longer duration of postvaccinal headache. In patients who had suffered ischemic stroke, headaches occurred an average of 1 day after receiving Comirnaty, 2.5 days after receiving Spikevax, and 7 days after receiving Jcovden. If the patients experienced intracranial hemorrhage as a complication, then headache occurred an average of 2 days after receiving Comirnaty, 6 days after receiving Spikevax, and 11 days after receiving the Jcovden vaccine. The main finding of the study by Garcia-Azorin et al. [31] was that a delayed onset of headache of more than 3 days indicated the onset of a serious cerebrovascular event, such as cerebral venous thrombosis, ischemic stroke, or intracranial hemorrhage.

**Figure 7:**
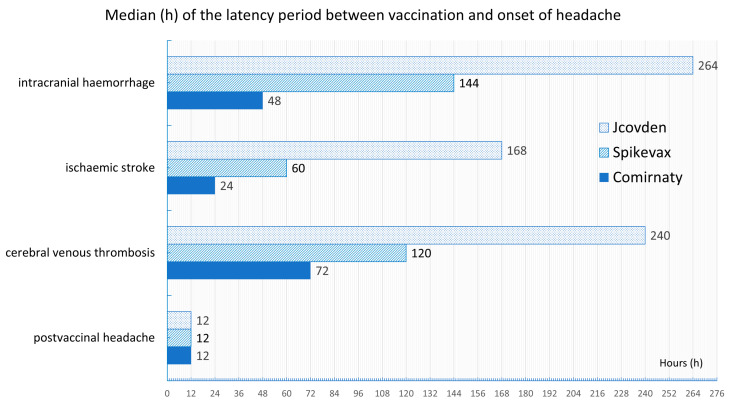
Median latency between vaccination against COVID-19 and headache onset. Combination of the data from this study for postvaccinal headache and the data from Garcia-Azorin et al. [31] for headaches in patients with cerebral venous thrombosis, ischemic stroke, and intracranial hemorrhage.

Figure 7 shows a comparison between the medians of the latency period between vaccination against COVID-19 and the onset of headache. The data from our study on postvaccinal headaches and the data from Garcia-Azorin et al. [31] on headaches in patients with cerebral venous thrombosis, ischemic stroke, and intracranial hemorrhage from the U.S. vaccination register are presented in combination.

Headaches are the fourth most common reason for emergency department visits [82,83]. Precise knowledge of the headache phenotype is essential in order for healthcare professionals to quickly identify the patients with life-threatening causes of new-onset headache who require emergency management.

On the other hand, these headaches must be differentiated from forms of headache that also cause a high level of suffering and require specialized treatment. The so-called “red flags” and “orange flags” for differentiating and recognizing threatening headaches must be identified [84]. These factors include the occurrence of new headaches that have never occurred before in this form (“headache like never before”), a lack of effectiveness in previously effective painkillers, an increasing worsening of headaches, a change in the phenotype of the headache, and the occurrence of previously unknown accompanying symptoms (fever, chills, neck stiffness, joint pain, neuropsychological disorders, etc.). Knowledge of the headache phenotype and its use in the differential diagnosis of different types of headaches is therefore of great clinical importance.

Recognizing delayed headache after COVID-19 vaccination requires the knowledge that the typical phenotype of headache involves the occurrence of headache within 1 day as part of the immune response. The present study documented onset within 1 day and described the complex phenotype of headache attributable to COVID-19 vaccination. The occurrence of delayed headache after vaccination against COVID-19 has now been included in the WHO guidelines for the clinical case management of thrombosis with thrombocytopenia syndrome (TTS) after COVID-19 vaccination [81].

Jameie et al. [33] retrospectively analyzed the characteristics of postvaccinal headaches in a cohort study in Iran. A total of 1,822 respondents completed a questionnaire on the characteristics of postvaccinal headaches. The prevalence rates of headache after the first, second, and third doses were 36.5%, 23.3%, and 21.7%, respectively. The authors stated that the headaches were mainly tension-type headaches (46.5%), followed by migraine-type headaches (3.61%). The headaches were mainly bilateral (69.7%), and the headache characteristics were mostly pressing (54.3%) and of medium intensity (51.0%). Headaches usually started within 10 h (4.0–24 h) after vaccination and lasted for 24 h (4–48 h). The authors cited a history of primary headache, post-COVID-19 headache or headache after a previous COVID-19 vaccination, fever, and vaccination with a vector vaccine as risk factors for postvaccinal headache. According to the authors’ data, primary headache and headache after COVID-19 vaccination should reduce the likelihood of prolonged headache after vaccination. Prolonged headaches after previous vaccinations, migraine-like headaches, and psychiatric disorders are thought to increase the risk of headaches after COVID-19 vaccination. This study did not adhere closely to the criteria of the International Headache Classification [37]; therefore, the generalizability of the results is limited. For example, questions were asked about “similarity” to the primary headache without explicitly recording the characteristics.

Raethke et al. [49] comprehensively examined adverse events associated with COVID-19 vaccines in seven European countries. As part of a multinational European collaboration, a prospective cohort study was conducted to monitor adverse events associated with COVID-19 vaccines. The participants completed questionnaires initially after vaccination and then six further questionnaires on self-reported adverse events over at least six months after the first dose of the COVID-19 vaccine. Additionally, 10 follow-up questionnaires were made available for one year. More than 117,791 individuals completed the study. Fatigue and headache were the most frequently reported systemic side effects. Pain at the injection site was the most common local reaction. Headache was reported by 53.3% of Vaxzevria recipients, 11.9% of Pfizer recipients, 49.6% of Janssen recipients, and 29.8% of Moderna recipients. Further characteristics of the headaches are not described.

Ekizoglu et al. [30] described the frequency and clinical characteristics of postvaccinal headaches in employees at a university hospital. A total of 1819 people who had been vaccinated with a vector vaccine were included in the study. A total of 30.6% of the participants reported postvaccinal headaches. A total of 25.9% reported headaches lasting more than three days. The headaches were mostly bilateral. The presence of primary headache was significantly associated with the occurrence of postvaccinal headache. Postvaccinal headaches are thought to occur mainly in women with pre-existing primary headaches, thyroid disease, headaches during COVID-19 vaccination, or headaches associated with influenza vaccination.

Ceccradi et al. [29] investigated the prevalence and clinical characteristics of postvaccinal headaches among patients in emergency departments. Emergency department visits and subsequent hospitalizations for new or worsening headache in the first 16 days after COVID-19 vaccination between January 2021 and January 2022 were recorded and compared with data from January 2019 to January 2020. Following the administration of a COVID-19 vaccination, the number of admissions to the emergency department due to headaches increased significantly. Approximately 14% of all patients who visited the emergency department due to headaches related to COVID-19 vaccination had to be hospitalized. No analyses of headache phenotypes were performed in the study by Ceccradi et al.

Atalar et al. [35] investigated the long-term course of postvaccinal headaches in 175 patients. This study analyzed different phenotypes of persistent or worsening headaches associated with COVID-19 vaccination. The study was able to differentiate two clusters. Patients with a history of primary headache presented frontal pain localization, throbbing pain, and more severe headache with migraine-like accompanying symptoms. The second group consisted of patients with longer headache durations (over one month) and a stabbing/pressing pain quality.

Castaldo et al. [28] conducted a systematic literature review on headaches after COVID-19 vaccination. Their analysis included 1.57 million participants, 94% of whom received the Biontech or Vaxzevria vaccine. Headache was the third most common adverse event and occurred in 22% of respondents after the first dose and in 29% after the second dose. The authors were unable to detect any differences between the mRNA-based vaccines and the vector vaccines.

Sekiguchi et al. [34] investigated the occurrence of postvaccinal headaches in patients with pre-existing headaches. The authors examined whether patients with pre-existing headaches were more likely to suffer from postvaccinal headaches. To this end, they conducted a study among nursing staff in a hospital. They categorized the sample into patients with migraines, patients with non-migraine-related headaches, and healthy controls. The frequency of postvaccinal headaches was significantly greater in the migraine group (69.2%) and in the group with non-migraine-related headaches (7.14%) than in the healthy control group (3.79%). The frequency of headaches was significantly greater after the second vaccination dose than after the first vaccination dose (4.56% versus 20.5%). The authors concluded that patients with a history of migraine or other headaches were more likely to experience postvaccinal headaches than healthy controls.

### 4.10. Limitations

This analysis has several limitations. Owing to the voluntary nature of participation in the study, it is possible that respondents with particularly severe headaches were more likely to participate. The resulting clinical headache phenotype could therefore describe particularly severe forms. We therefore tried to capture the headache phenotype as comprehensively as possible by recruiting a very large number of participants internationally. No definitive conclusions can be drawn regarding gender differences. As the population of vaccinated individuals is not known and participation in the study was voluntary, it is possible that a disproportionate number of women participated in the study. As headaches occur very frequently regardless of vaccination status, the data could be biased due to the occurrence of headaches unrelated to the COVID-19 vaccine. However, the resulting headache phenotype is not congruent with either migraines or tension-type headaches. It is possible that the clinical symptoms of these two common primary headache forms were included in the responses and overlapped with the post-COVID-19 vaccination headache phenotype. Nevertheless, the described symptom complex of headache after COVID-19 vaccination is clearly distinct from the abovementioned primary headaches. The possible influence of analgesic treatment of headaches is not known. This study did not investigate the prevalence of postvaccinal headaches. Phase 3 clinical trials of various vaccine candidates provided detailed information on this topic by recording the frequency of possible side effects of the vaccination [59,60,61,64,66,67,68,69,73,74,75,76,85]. On the other hand, no detailed information on the clinical phenotype was reported in these studies. The questionnaire did not contain any mandatory questions. Therefore, complete information is not available for the entire sample.

### 4.11. Pathological Mechanisms

The pathological mechanisms behind postvaccinal headaches have not yet been elucidated. It remains unclear whether the spike protein, which is synthesized intracellularly with the help of the mRNA supplied by the vaccine, is responsible for the headaches or whether the headaches are due to the immune reaction triggered by this protein [50,66,86,87,88,89,90,91]. The intracellular formation of the spike protein and the resulting immune response could be directly related to the development of the described headache phenotype, including the accompanying symptoms of tiredness, fatigue, muscle pain, dizziness, poor concentration, chills, and fever. The vaccine-induced SARS-CoV-2 spike protein could cause short-term inflammatory and other pathological changes in the brain in the area of the craniocerebral axis [92]. As the process is time-limited, symptoms usually subside after 48 h but can also persist in some individual cases.

This process can activate inflammatory substances such as nitric oxide, prostaglandins, and cytokines [86,87]. Large quantities of proinflammatory cytokines can be released during COVID-19 infection. According to our results, fever is a prominent concomitant symptom, especially among individuals who have received Vaxzevria. It can therefore be assumed that inflammatory mediators are also involved in the development of postvaccinal headaches. Pre-existing primary headaches such as migraines can lead to an increased duration and pain intensity in postvaccinal headaches. The existing sensitization with hyperexcitability of the trigeminovascular neurons in primary headaches may cause an increase in pain sensitivity, which is relevant to the intensification of postvaccinal headaches. Accordingly, a study by van der Arend et al. [93] suggested that vaccination against COVID-19 may be associated with an increase in migraine duration in migraine patients. These results may indicate that the involvement of inflammatory mediators in the pathophysiology of migraine may influence the intrinsic threshold for approaching attacks.

A person’s pre-existing conditions and immune system can be decisive in the course of COVID-19 disease and the possible development of postvaccinal headaches. Genetic make-up can also directly influence how much someone is at risk and how severe the disease is. This includes factors such as the cytokine and interleukin genes responsible for proinflammatory and immunomodulatory responses, as well as the genes thought to be responsible for the progression and complications of COVID-19 [94].

## 5. Conclusions

Headaches due to COVID-19 vaccination have an extensive and characteristic symptom complex. These characteristics can vary across different vaccines. The complex accompanying symptoms and the temporal and spatial characteristics of postvaccinal headaches compose the headache phenotype. The findings of this study have important clinical implications in the differentiation of postvaccinal headache from other primary and secondary headaches. Based on these results, new diagnostic criteria for this form of headache can be developed.

## Data Availability

The datasets generated during and/or analyzed during the current study are available from the corresponding author upon reasonable request.

## Supplementary Materials

The following supporting information can be downloaded at: https://www.mdpi.com/article/10.3390/vaccines13020113/s1, Supplementary File S1. Table S1: Vaccines and their mode of action in phase 1 to phase 4 worldwide (as of 30 March 2023) [22]. Table S2. Types. modes of action and ingredients of COVID-19 vaccines [21]. Table S3. Comparison of the age of the vaccinated individuals between vaccines (significance ANOVA: *p* = 0.735). Table S4: Gender distribution between vaccines (Chi-square test: *p* < 0.001). Table S5. Comparison of latency (h) between the administration of the vaccination and the onset of headache between the vaccines (ANOVA: *p* < 0.001). Table S6. Comparison of the latency between the administration of the vaccine and the onset of headache between the vaccines. Post hoc analysis with the Scheffé test. Table S7. Frequency distribution of the temporal pattern of headache onset between vaccines (Chi-square test: *p* < 0.001). Table S8: Frequency distribution of the preferred time of day for the occurrence of headaches between vaccines (chi-square test: *p* < 0.001). Table S9. Frequency distribution of side locations of headache occurrence between vaccines (Chi-square test: *p* < 0.001). Table S10. Frequency distribution of the incidence of headaches between vaccines (chi-square test: *p* < 0.001). Table S11: Frequency distributions of pain radiation between vaccines (chi-square test: *p* < 0.001). Table S12. Frequency distributions of headache characteristics between vaccines (chi-square test: *p* < 0.001). Table S13. Comparison of headache intensities between vaccines (ANOVA: *p* < 0.001). Table S14. Comparison of headache intensities between vaccines: post hoc individual comparisons via the Scheffé test. Table S15. Frequency distributions of the effects of routine physical activity on headaches between vaccines (chi-square test; *p* < 0.001). Table S16. Frequency distributions of the effects of body position on headaches between vaccines (chi-square test; *p* < 0.001). Table S17. Frequency distribution of migraine-like concomitant symptoms of headache between vaccines (Chi-square test; *p* < 0.001). Table S18. Concomitant symptoms reported by more than 10% of respondents between vaccines (chi-square test; *p* < 0.001).
