# Supplementary material for: Comparison of Phenotypes of Headaches After COVID-19 Vaccinations Differentiated According to the Vaccine Used
Source: Vaccines (Basel). 2025 Jan 23;13(2):113. doi: 10.3390/vaccines13020113 (PMC11861871; doi:10.3390/vaccines13020113)
Supplement: Supplementary file 1 [file vaccines-13-00113-s001.zip › vaccines-3423416-supplementary.pdf]

Supplementary File S1

Tables

Table S1: Vaccines and their mode of action in phase 1 to phase 4 worldwide (as of 30 March 2023) [22]

**Operating principle**		**Vaccine candidates**	
		**N**	**%**
**PS**	Protein subunit	59	32%
**VVnr**	Viral Vector (nonreplicating)	25	14%
**DNA**	DNA	17	9%
**IV**	Inactivated Virus	22	12%
**RNA**	RNA	43	24%
**VVr**	Viral Vector (replicating)	4	2%
**VLP**	Virus Like Particle	7	4%
**VVr + APC**	VVr + Antigen Presenting Cell	2	1%
**LAV**	Live Attenuated Virus	2	1%
**VVnr + APC**	VVnr + Antigen Presenting Cell	1	1%
**BacAg-SpV**	Bacterial antigen-spore expression vector	1	1%

Table S2. Types. modes of action and ingredients of COVID-19 vaccines [21]

Type	Principle	Ingredients
Virus components	Protein subunit	Isolated and purified viral proteins
	Virus-like Particles (VLP)	Nongenetic viral proteins that resemble the virus structure
	DNA-based	Genetic material for the production of viral proteins
	RNA-based	
	Non-replicating Viral Vector	Genetic material packed into a harmless virus that cannot replicate
	Replicating Viral Vector	Genetic material packed into a harmless virus that can replicate
Entire virus	Inactivated	Killed virus
	Live-attenuated	Attenuated virus

Table S3. Comparison of the age of the vaccinated individuals between vaccines (significance ANOVA: p=0.735)

		Age (years)	
Vaccine	N	Arith. Mean	Std. dev.
Comirnaty (BioNTech)	6309	41.165	27.2030
Jcovden (Johnson & Johnson)	130	38.277	10.9891
Sputnik V (Gamelaya)	33	43.424	10.6332
Covilo (Sinopharm)	59	42.593	8.7319
Spikevax (Moderna)	1226	41.060	12.4881
Vaxzevria (AstraZeneca)	3121	41.458	13.2946
Convidecia (CanSino Biologics)	11	37.455	10.9760
Total	10889	41.214	22.3464

Table S4: Gender distribution between vaccines (Chi-square test: p<0.001)

	Comirnaty (BioNTech)	Jcovden (Johnson & Johson)	Sputnik V (Gamelaya)	Covilo (Sinopharm)	Spikevax (Moderna)	Vaxzevria (AstraZeneca)	Convidecia (CanSino Biologics)	Total
Men	19.9%	31.5%	27.3%	41.9%	18.3%	19.0%	27.3%	19.7%
Women	80.0%	68.5%	72.7%	58.1%	81.5%	80.9%	72.7%	80.2%
Miscellaneous	0.1%	0.0%	0.0%	0.0%	0.2%	0.1%	0.0%	0.1%

Table S5. Comparison of latency (h) between the administration of the vaccination and the onset of headache between the vaccines (ANOVA: p < 0.001)

	N	Arith. mean	Std. dev.	Std. error	95% confidence interval	
					Lower limit	Upper limit
Jcovden (Johnson &	155	30.01	47.74	3.835	22.44	37.59
Comirnaty (BioNTech)	7473	26.06	37.48	.434	25.21	26.91
Spikevax (Moderna)	1438	22.02	34.25	.903	20.25	23.79
Covilo (Sinopharm)	119	20.54	28.98	2.657	15.28	25.80
Convidecia (CanSino	14	19.18	43.53	11.63	-5.96	44.31
Vaxzevria	3616	17.98	28.03	.466	17.06	18.89
Sputnik V (Gamelaya)	43	17.07	20.12	3.069	10.88	23.26
Total	1285	23.29	34.93	.308	22.69	23.90

Table S6. Comparison of the latency between the administration of the vaccine and the onset of headache between the vaccines. Post hoc analysis with the Scheffé test

Vaccine	Individual comparison	Mean value	Std. error	Sig.
Comirnaty (BioNTech)	Jcovden (Johnson &	-3.955	2.820	.923
	Sputnik V (Gamelaya)	8.988	5.315	.826
	Covilo (Sinopharm)	5.518	3.211	.815
	Spikevax (Moderna)	4.039	1.001	.012
	Vaxzevria (AstraZeneca)	8.082	.704	<.0
	Convidecia (CanSino	6.879	9.296	.997
Jcovden (Johnson & Johnson)	Comirnaty (BioNTech)	3.955	2.820	.923
	Sputnik V (Gamelaya)	12.943	5.990	.587
	Covilo (Sinopharm)	9.473	4.235	.544
	Spikevax (Moderna)	7.994	2.938	.285
	Vaxzevria (AstraZeneca)	12.037	2.850	.007
	Convidecia (CanSino	10.834	9.698	.974
Sputnik V (Gamelaya)	Comirnaty (BioNTech)	-8.988	5.315	.826
	Jcovden (Johnson &	-12.943	5.990	.587
	Covilo (Sinopharm)	-3.471	6.183	.999
	Spikevax (Moderna)	-4.949	5.378	.991
	Vaxzevria (AstraZeneca)	-.906	5.331	1.00
	Convidecia (CanSino	-2.109	10.69	1.00
Covilo (Sinopharm)	Comirnaty (BioNTech)	-5.518	3.211	.815
	Jcovden (Johnson &	-9.473	4.235	.544
	Sputnik V (Gamelaya)	3.471	6.183	.999
	Spikevax (Moderna)	-1.478	3.315	1.00
	Vaxzevria (AstraZeneca)	2.565	3.238	.996
	Convidecia (CanSino	1.362	9.819	1.00
Spikevax (Moderna)	Comirnaty (BioNTech)	-4.039	1.001	.012
	Jcovden (Johnson &	-7.994	2.938	.285
	Sputnik V (Gamelaya)	4.949	5.378	.991
	Covilo (Sinopharm)	1.478	3.315	1.00
	Vaxzevria (AstraZeneca)	4.043	1.083	.031
	Convidecia (CanSino	2.840	9.333	1.00
Vaxzevria (AstraZeneca)	Comirnaty (BioNTech)	-8.082	.704	<.0
	Jcovden (Johnson &	-12.037	2.850	.007
	Sputnik V (Gamelaya)	.906	5.331	1.00
	Covilo (Sinopharm)	-2.565	3.238	.996
	Spikevax (Moderna)	-4.043	1.083	.031
	Convidecia (CanSino	-1.203	9.305	1.00
Convidecia (CanSino Biologics)	Comirnaty (BioNTech)	-6.879	9.296	.997
	Jcovden (Johnson &	-10.834	9.698	.974
	Sputnik V (Gamelaya)	2.109	10.69	1.00
	Covilo (Sinopharm)	-1.362	9.819	1.00
	Spikevax (Moderna)	-2.840	9.333	1.00
	Vaxzevria (AstraZeneca)	1.203	9.305	1.00

Table S7. Frequency distribution of the temporal pattern of headache onset between vaccines (Chi-square test: P<0.001). The color indicates maximum values.

	Comirnaty (BioNTech)	Jcovden (Johnson & Johnson)	Sputnik V (Gamelaya)	Covilo (Sinopharm)	Spikevax (Moderna)	Vaxzevria (AstraZeneca)	Convidecia (CanSino Bioloics)	Total
Undulating	19.9%	20.8%	30.2%	35.9%	18.9%	16.4%	21.4%	19.0%
Creeping	59.5%	53.2%	44.2%	48.7%	57.4%	62.3%	50.0%	59.8%
Peracute	20.5%	26.0%	25.6%	15.4%	23.6%	21.2%	28.6%	21.2%

Table S8: Frequency distribution of the preferred time of day for the occurrence of headaches between vaccines (chi-square test: p<0.001). The color indicates maximum values.

	Comirnaty (BioNTech)	Jcovden (Johnson & Johnson)	Sputnik V (Gamelaya)	Covilo (Sinopharm)	Spikevax (Moderna)	Vaxzevria (AstraZeneca)	Convidecia (CanSino Biologics)	Total
Night	8.4%	9.0%	25.6%	16.5%	8.1%	10.9%	15.4%	9.2%
Changing	23.4%	26.5%	25.6%	26.1%	23.5%	22.0%	15.4%	23.2%
Day	28.4%	20.0%	25.6%	36.5%	21.6%	21.0%	30.8%	25.5%
Permanent	39.8%	44.5%	23.3%	20.9%	46.8%	46.1%	38.5%	42.2%

Table S9. Frequency distribution of side locations of headache occurrence between vaccines (Chi-square test: p<0.001). The color indicates maximum values.

	Comirnaty (BioNTech)	Jcovden (Johnson & Johnon)	Sputnik V (Gamelaya)	Covilo (Sinopharm)	Spikevax (Moderna)	Vaxzevria (AstraZeneca)	Convidecia (CanSino Biologics)	Total
Unilaterally	13.0%	11.3%	2.8%	15.6%	12.8%	9.5%	9.1%	12.0%
Unilateral	24.3%	19.0%	33.3%	27.3%	23.9%	17.3%	36.4%	22.3%
Bilateral	62.8%	69.7%	63.9%	57.1%	63.3%	73.2%	54.5%	65.7%

Table S10. Frequency distribution of the incidence of headaches between vaccines (chi-square test: p<0.001). The color indicates maximum values.

	Comirnaty (BioNTech)	Jcovden (Johnson & Johson)	Sputnik V (Gamelaya)	Covilo (Sinopharm)	Spikevax (Moderna)	Vaxzevria (AstraZeneca)	Convidecia (CanSino Biologics)	Total
Neck right	19.7%	20.0%	14.0%	6.7%	22.0%	18.9%	0.0%	19.6%
Neck left	20.1%	21.3%	14.0%	8.4%	22.3%	19.4%	0.0%	20.0%
Occipital right	30.2%	41.9%	32.6%	12.6%	31.7%	31.1%	21.4%	30.6%
Occipital left	30.4%	39.4%	27.9%	16.0%	32.0%	30.5%	21.4%	30.5%
Temporal right	38.2%	45.8%	20.9%	11.8%	40.0%	38.6%	21.4%	38.2%
Temporal left	38.5%	41.3%	20.9%	13.4%	39.7%	37.5%	28.6%	38.1%
Forehead right	44.3%	45.8%	27.9%	23.5%	45.5%	46.5%	42.9%	44.8%
Forehead left	44.0%	47.1%	30.2%	23.5%	44.4%	46.5%	64.3%	44.6%
Skullcap	25.2%	29.0%	23.3%	29.4%	25.1%	25.9%	7.1%	25.4%
Retroorbital	30.1%	38.1%	37.2%	16.8%	33.0%	30.0%	14.3%	30.4%

Table S11: Frequency distributions of pain radiation between vaccines (chi-square test: p<0.001). The color indicates maximum values.

	Comirnaty (BioNTech)	Jcovden (Johnson & Johnon)	Sputnik V (Gamelaya)	Covilo (Sinopharm)	Spikevax (Moderna)	Vaxzevria (AstraZeneca)	Convidecia (CanSino Bioloics)	Total
To forehead and	24.5%	18.2%	11.1%	20.5%	28.5%	24.3%	18.2%	24.7%
to neck and	20.8%	25.2%	13.9%	12.8%	20.4%	17.0%	45.5%	19.7%
No radiation	54.8%	56.6%	75.0%	66.7%	51.1%	58.7%	36.4%	55.6%

Table S12. Frequency distributions of headache characteristics between vaccines (chi-square test: p<0.001). The color indicates maximum values.

	Comirnaty (BioNTech)	Jcovden (Johnson & Johson)	Sputnik V (Gamelaya)	Covilo (Sinopharm)	Spikevax (Moderna)	Vaxzevria (AstraZeneca)	Convidecia (CanSino Biolgics)	Total
Tearing	3.3%	3.9%	4.7%	0.0%	3.9%	2.8%	0.0%	3.2%
Cutting	3.4%	3.2%	7.0%	0.0%	4.2%	4.0%	7.1%	3.7%
Hot	5.8%	3.2%	11.6%	5.0%	7.4%	5.9%	0.0%	6.0%
Cramping	6.3%	11.6%	11.6%	7.6%	6.9%	4.8%	7.1%	6.1%
Burning	6.6%	9.7%	2.3%	2.5%	7.2%	4.6%	0.0%	6.1%
Drilling	12.5%	16.8%	9.3%	5.0%	14.0%	11.9%	7.1%	12.5%

Hammering	15.3%	16.8%	7.0%	5.0%	17.2%	16.6%	7.1%	15.7%
Pulling	19.1%	20.0%	11.6%	3.4%	17.7%	16.3%	28.6%	18.0%
Throbbing	20.0%	23.2%	11.6%	16.0%	22.9%	19.0%	21.4%	20.0%
Pulsating	23.2%	21.3%	23.3%	21.0%	27.5%	21.1%	7.1%	23.0%
Stinging	24.1%	26.5%	16.3%	5.0%	26.3%	21.9%	21.4%	23.5%
Dull	42.9%	43.2%	30.2%	21.8%	39.8%	44.4%	7.1%	42.7%
Tearing	3.3%	3.9%	4.7%	0.0%	3.9%	2.8%	0.0%	3.2%

Table S13. Comparison of headache intensities between vaccines (ANOVA: p<0.001)

	N	Mean value	Std. dev.	Std. error	95% confidence interval	
					Lower limit	Upper limit
Spikevax (Moderna)	1306	3.72	0.872	0.024	3.67	3.77
Jcovden (Johnson & Johnson)	138	3.63	0.88	0.075	3.48	3.78
Comirnaty (BioNTech)	6760	3.56	0.884	0.011	3.54	3.58
Vaxzevria (AstraZeneca)	3290	3.56	0.908	0.016	3.53	3.59
Convidecia (CanSino Biologics)	11	3.55	1.128	0.34	2.79	4.30
Sputnik V (Gamelaya)	36	3.31	1.037	0.173	2.95	3.66
Covilo (Sinopharm)	74	3.05	0.89	0.103	2.85	3.26
Total	11615	3.57	.892	.008	3.56	3.59

Table S14. Comparison of headache intensities between vaccines: post hoc individual comparisons via the Scheffé test

Vaccine	Individual comparison	Mean value difference	Std. error	Sig.
Comirnaty (BioNTech)	Jcovden (Johnson & Johnson)	-.071	.077	.991
	Sputnik V (Gamelaya)	.254	.149	.819
	Covilo (Sinopharm)	.506	.104	<.0
	Spikevax (Moderna)	-.162	.027	<.0
	Vaxzevria (AstraZeneca)	.000	.019	1.00
	Convidecia (CanSino	.014	.269	1.00
Jcovden (Johnson & Johnson)	Comirnaty (BioNTech)	.071	.077	.991
	Sputnik V (Gamelaya)	.325	.167	.703
	Covilo (Sinopharm)	.576	.128	.003
	Spikevax (Moderna)	-.091	.080	.972
	Vaxzevria (AstraZeneca)	.071	.077	.991
	Convidecia (CanSino	.085	.279	1.00
Sputnik V (Gamelaya)	Comirnaty (BioNTech)	-.254	.149	.819
	Jcovden (Johnson & Johnson)	-.325	.167	.703
	Covilo (Sinopharm)	.252	.181	.926
	Spikevax (Moderna)	-.416	.150	.266
	Vaxzevria (AstraZeneca)	-.254	.149	.822
	Convidecia (CanSino	-.240	.307	.996

Covilo (Sinopharm)	Comirnaty (BioNTech)	-.506	.104	<.0
	Jcovden (Johnson & Johnson)	-.576	.128	.003
	Sputnik V (Gamelaya)	-.252	.181	.926
	Spikevax (Moderna)	-.667	.106	<.0
	Vaxzevria (AstraZeneca)	-.505	.105	<.0
	Convidecia (CanSino	-.491	.288	.819
Spikevax (Moderna)	Comirnaty (BioNTech)	.162	.027	<.0
	Jcovden (Johnson & Johnson)	.091	.080	.972
	Sputnik V (Gamelaya)	.416	.150	.266
	Covilo (Sinopharm)	.667	.106	<.0
	Vaxzevria (AstraZeneca)	.162	.029	<.0
	Convidecia (CanSino	.176	.270	.999
Vaxzevria (AstraZeneca)	Comirnaty (BioNTech)	.000	.019	1.00
	Jcovden (Johnson & Johnson)	-.071	.077	.991
	Sputnik V (Gamelaya)	.254	.149	.822
	Covilo (Sinopharm)	.505	.105	<.0
	Spikevax (Moderna)	-.162	.029	<.0
	Convidecia (CanSino	.014	.269	1.00
Convidecia (CanSino Biologics)	Comirnaty (BioNTech)	-.014	.269	1.00
	Jcovden (Johnson & Johnson)	-.085	.279	1.00
	Sputnik V (Gamelaya)	.240	.307	.996
	Covilo (Sinopharm)	.491	.288	.819
	Spikevax (Moderna)	-.176	.270	.999
	Vaxzevria (AstraZeneca)	-.014	.269	1.00

Table S15. Frequency distributions of the effects of routine physical activity on headaches between vaccines (chi-square test; p < 0.001). The color indicates maximum values.

	Comirnaty (BioNTech)	Jcovden (Johnson & Johson)	Sputnik V (Gamelaya)	Covilo (Sinopharm)	Spikevax (Moderna)	Vaxzevria (AstraZeneca)	Convidecia (CanSino Bioloics)	Total
No effect	46.2%	42.2%	52.9%	54.1%	41.8%	46.1%	45.5%	45.7%
soothing	8.6%	11.1%	8.8%	16.2%	6.0%	6.4%	18.2%	7.8%
reinforcing	45.2%	46.7%	38.2%	29.7%	52.2%	47.6%	36.4%	46.5%

Table S16. Frequency distributions of the effects of body position on headaches between vaccines (chi-square test; p < 0.001). The color indicates maximum values.

	Comirnaty (BioNTech)	Jcovden (Johnson & Johson)	Sputnik V (Gamelaya)	Covilo (Sinopharm)	Spikevax (Moderna)	Vaxzevria (AstraZeneca)	Convidecia (CanSino Biologics)	Total
Independent of	63.2%	57.8%	52.9%	49.3%	61.3%	64.5%	50.0%	63.1%
Better when lying	29.2%	30.4%	38.2%	49.3%	29.9%	28.5%	50.0%	29.3%
Better when standing	7.6%	11.9%	8.8%	1.4%	8.8%	7.0%	0.0%	7.6%

Table S17. Frequency distribution of migraine-like concomitant symptoms of headache between vaccines (Chi-square test; p < 0.001). The color indicates maximum values.

	Comirnaty (BioNTech)	Jcovden (Johnson & Johson)	Sputnik V (Gamelaya)	Covilo (Sinopharm)	Spikevax (Moderna)	Vaxzevria (AstraZeneca)	Convidecia (CanSino Biologics)	Total
Vomiting	4.5%	5.8%	9.3%	5.0%	6.5%	4.7%	0.0%	4.9%
Osmophobia	6.2%	7.7%	7.0%	3.4%	6.8%	4.4%	0.0%	5.8%
Loss of appetite	19.4%	25.8%	30.2%	11.8%	26.9%	25.5%	35.7%	22.1%
Nausea	34.6%	38.7%	25.6%	15.1%	38.0%	32.6%	28.6%	34.3%
Photophobia	37.9%	38.1%	25.6%	12.6%	39.1%	37.2%	21.4%	37.6%
Phonophobia	39.3%	40.6%	37.2%	16.0%	40.4%	39.2%	35.7%	39.2%

Table S18. Concomitant symptoms reported by more than 10% of respondents between vaccines (chi-square test; p < 0.001). The color indicates maximum values.

	Comirnaty (BioNTech)	Jcovden (Johnson & Johnon)	Sputnik V (Gamelaya)	Covilo (Sinopharm)	Spikevax (Moderna)	Vaxzevria (AstraZeneca)	Convidecia (CanSino Biologics)	Total
Reddening of the	8.6%	5.2%	9.3%	2.5%	17.5%	10.9%	0.0%	10.1%
Fears	12.2%	14.8%	14.0%	6.7%	12.1%	9.2%	7.1%	11.3%
Irritability	12.2%	14.8%	14.0%	6.7%	12.1%	9.2%	7.1%	11.3%
Inner restlessness	13.0%	16.1%	11.6%	5.0%	13.8%	11.8%	0.0%	12.7%
Sweating	14.1%	21.9%	27.9%	7.6%	20.0%	17.5%	7.1%	15.8%
Loss of appetite	14.0%	21.3%	20.9%	4.2%	19.3%	20.1%	21.4%	16.4%
Fever	9.5%	14.8%	18.6%	2.5%	19.9%	30.7%	14.3%	16.6%
Phonophobia	18.8%	20.6%	11.6%	5.9%	20.0%	17.8%	7.1%	18.5%

Joint pain	14.1%	17.4%	16.3%	5.9%	20.7%	27.0%	14.3%	18.5%
Photophobia	18.9%	17.4%	18.6%	5.9%	19.0%	18.8%	7.1%	18.7%
Chills	13.9%	20.0%	25.6%	2.5%	25.0%	36.3%	7.1%	21.4%
Concentration	22.4%	28.4%	20.9%	2.5%	23.9%	21.4%	0.0%	22.1%
Physical weakness	22.8%	24.5%	32.6%	16.0%	27.7%	30.4%	21.4%	25.5%
Neck pain	25.9%	23.2%	16.3%	6.7%	29.0%	24.2%	7.1%	25.5%
Muscle pain	21.2%	18.7%	27.9%	10.9%	31.1%	35.3%	14.3%	26.1%
Dizziness	27.4%	29.0%	27.9%	12.6%	29.1%	29.3%	28.6%	28.0%
Exhaustion	33.4%	40.0%	25.6%	6.7%	37.5%	37.9%	0.0%	34.9%
Tiredness	45.4%	47.1%	48.8%	18.5%	48.0%	49.0%	21.4%	46.4%
